# Supplementary material for: The Nβ motif of NaTrxh directs secretion as an endoplasmic reticulum transit peptide and variations might result in different cellular targeting
Source: PLoS One. 2023 Oct 12;18(10):e0287087. doi: 10.1371/journal.pone.0287087 (PMC10569557; doi:10.1371/journal.pone.0287087)
Supplement: S2 Table — (DOCX) [file pone.0287087.s002.docx]

**S2 Table.** **Primers used for the different constructs generated for transient expression assays in onion epidermal cells.**

| **Primer name**^a^ | **Sequence (5’ – 3’)**^b^ |
| --- | --- |
| Nβ(-3)-F | CACCATGGAATCAGGATCGTCGTCAGAACCG |
| Nβ(-6)-F | CACCATGTCGTCGTCAGAACCG |
| NaT-R | TTGGACATGAAATTTAGTTCGATAATTACTAGCAGC |
| GFP-R | TCACACGTGGTGGTGGTGGTGGTGGCTAGC |
| Nβ-F | CACCATGGCAGAGGCAGAATCAGGATCGTCGTCAGAACCG |
| Nβ(S5G)-F | CTGACGACGATCCTCCTTCTGCCTCTGCCATGGT |
| Nβ(S5G)-R | ACCATGGCAGAGGCAGAAGGAGGATCGTCGTCAG |
| Nβ(S8E)-F | CAACCCTTCGGTTCTGACTCCGATCCTGATTCTGCCT |
| Nβ(S8E)-R | AGGCAGAATCAGGATCGGAGTCAGAACCGAAGGGTG |
| Nβ(S8D)-F | CCCACCCTTCGGTTCTGAGTCCGATCCTGATTCTGCCTC |
| Nβ(S8D)-R | GAGGCAGAATCAGGATCGGACTCAGAACCGAAGGGTGGG |
| Nβ(S8A)-F | CCTTCGGTTCTGACGCCGATCCTGATTCTGC |
| Nβ(S8A)-R | GCAGAATCAGGATCGGCGTCAGAACCGAAGG |
| Nβ(S9A)-F | CACCCTTCGGTTCTGCCGACGATCCTGATTC |
| Nβ(S9A)-R | GAATCAGGATCGTCGGCAGAACCGAAGGGTG |
| NaTΔαβ-F | *GGTACC*ATGTCGCGTGTGATTGCTTTTC |
| NaT-Nβ-R1 | TCCTGATTCTGCCTCTGCTTGGACATGAAATTTAGTTCGATAATTAC |
| NaT-Nβ-R2 | *GAATTC*GCCGGTTCTGACGACGATCCTGATTCTGCCTCTGC |
| GFP-F | CGC*GGATCC*ACCATGGGAAGTAAAGGAGAAGAACTTTTCAC |
| GFP-Nβ-R1 | TCCTGATTCTGCCTCTGCTTTGTATAGTTCATCCATGCCATGTG |
| GFP-Nβ-R2 | CCG*GAATTC*TTACGGTTCTGACGACGATCCTGATTCTGCCTCTGC |
| Nβinv-F1 | ATCGTCGGGATCAGAAGCAGAGGCAAAGGGTGGGCGCGCCGAC |
| GFP-R2 | CCG*GAATTC*TTATTTGTATAGTTCATCCATGCCATG |
| Nβinv-F2 | CGC*GGATCC*ACCATGCCGGAATCATCGTCGGGATCAGAAGCAG |
| NβKDEL-R | CCG*GAATTC*TTAAAGCTCATCTTTCGGTTCTGACGACGATC |
| pENTR4-F | GCGTTTCTACAAACTCTTCC |
| pENTR4-R | TTACTGTTTATGTAAGCAGACAG |

a. F = forward primers; R = reverse primers.

b. Highlighted in black with white letters: point mutations to replace the original codon for the desired one; italics: restriction sites (*Bam*-HI and *Eco*-RI).
